# Job strain and unhealthy lifestyle: results from the baseline cohort study, Brazilian Longitudinal Study of Adult Health (ELSA-Brasil)

**DOI:** 10.1186/s12889-015-1626-4

**Published:** 2015-03-31

**Authors:** Rosane Härter Griep, Aline Araújo Nobre, Márcia Guimarães de Mello Alves, Maria de Jesus Mendes da Fonseca, Letícia de Oliveira Cardoso, Luana Giatti, Enirtes Caetano Prates Melo, Susanna Toivanen, Dóra Chor

**Affiliations:** Laboratory of Health and Environment Education, Oswaldo Cruz Institute, Oswaldo Cruz Foundation, Avenida Brasil, 4365, Manguinhos, Rio de Janeiro, 21040-360 Brazil; Scientific Computing Program (PROCC), Fundação Oswaldo Cruz, Rio de Janeiro, RJ Brazil; Departamento de Planejamento em Saúde, Instituto de Saúde da Comunidade, Universidade Federal Fluminense, Niterói, RJ Brazil; National School of Public Health, Fundação Oswaldo Cruz, Rio de Janeiro, RJ Brazil; School of Nutrition, Universidade Federal de Ouro Preto, Belo Horizonte, MG Brazil; Centre for Health Equity Studies | CHESS, Stockholm University/Karolinska Institutet, Stockholm, Sweden

**Keywords:** Brazil, Job strain, Occupational health, Physical activity, Smoking, Epidemiology, Cross-sectional analysis

## Abstract

**Background:**

Unhealthy lifestyle choices, such as smoking and sedentary behavior, are among the main modifiable risk factors for chronic non-communicable diseases. The workplace is regarded as an important site of potential health risks where preventive strategies can be effective. We investigated independent associations among psychosocial job strain, leisure-time physical inactivity, and smoking in public servants in the largest Brazilian adult cohort.

**Methods:**

We conducted a cross-sectional analysis of baseline data from the Brazilian Longitudinal Study of Adult Health (ELSA-Brasil)—a multicenter prospective cohort study of civil servants. Our analytical samples comprised 11,779 and 11,963 current workers for, respectively, analyses of job strain and leisure-time physical activity and analyses of job strain and smoking. Job strain was assessed using the Brazilian version of the Swedish Demand-Control-Support Questionnaire; physical activity was evaluated using a short form of the International Physical Activity Questionnaire. We also examined smoking status and number of cigarettes smoked per day. The association reported in this paper was assessed by means of multinomial and logistic regression, stratified by sex.

**Results:**

Among men, compared with low-strain activities (low demand and high control), job strain showed an association with physical inactivity (odds ratio [OR] = 1.34; 95% confidence interval [CI] = 1.09–1.64) or with the practice of physical activities of less than recommended duration (OR = 1.44; 95% CI = 1.15–1.82). Among women, greater likelihood of physical inactivity was identified among job-strain and passive-job groups (OR = 1.47; 95% CI = 1.22–1.77 and OR = 1.42; 95% CI = 1.20–1.67, respectively). Greater control at work was a protective factor for physical inactivity among both men and women. Social support at work was a protective factor for physical inactivity among women, as was smoking for both genders. We observed no association between demand or control dimensions and smoking.

**Conclusions:**

Job strain, job control, and social support were associated with physical activity. Social support at work was protective of smoking. Our results are comparable to those found in more developed countries; they provide additional evidence of an association between an adverse psychosocial work environment and health-related behaviors.

## Background

Such behaviors as smoking and physical inactivity are among the main modifiable risk factors for chronic non-communicable diseases [1,2]. The workplace, where people spend most of their adult lives, is recognized as an important context for identifying potential health risks and implementing effective preventive strategies [3]. This conception underlies proposals that aim to promote healthy habits by reducing workplace health risks [4]—particularly strategies to support physical activity and providing the means to reduce barriers to engaging in such activity [5,6]. In addition, various measures to restrict the sale and consumption of tobacco, among them smoke-free areas (including workplaces), have been related to a substantial decline in smoking among both men and women [7].

Among work-related factors, psychosocial strain is considered one of the most important occupational risk factors for cardiovascular diseases and mental health disorders. Psychosocial strain entails high costs for employers and social security systems [8].

The Demand-Control Model, developed by Karasek [9], has been widely used to measure psychosocial strain at work. The model considers the interaction of two components: psychological demands (pace and intensity of work) and control (workers’ autonomy and job skill requirements). Work activities where the demand level is high and control low are considered “high strain”, and they are consequently associated with physical or psychological illness. Work with a combination of low demand and high control is regarded as “low strain”. “Active jobs”—in situations where both demand and control are simultaneously high—are related to active attitudes and the development of new patterns of behavior, both at work and outside. Lastly, low demand combined with low control characterizes “passive work”, which is related to a lessening of overall activity and reduced self-efficacy, resulting in more passive lifestyles [4,9]. A third dimension was later introduced into the original model—perceived social support at work; with this, social integration, confidence in the group, and obtaining colleagues’ and superiors’ help in performing tasks can all act protectively against the corrosive effects of work on health [10].

Several studies have related psychosocial characteristics of the work environment with cardiovascular outcomes under two main hypotheses: (1) direct effects on the immune, biological, and hormone systems; (2) indirect effects—the influence of the work environment on behavior in mediating a causal pathway between the psychosocial environment and health outcomes [11,12]. Some authors [11-13] argue that psychosocial job strain can be one of the factors contributing to the adoption or maintenance of health-related behavior. Work activities that lead, for example, to fatigue are considered to reinforce sedentary habits [14], and they hinder cessation of smoking or reinforce the consumption of an excessive number of cigarettes [15].

Studies conducted following this line of investigation have thus far produced inconclusive results [4,11,16], partly owing to small sample sizes, study design, and adopting different measures of job strain [16,17]. Some studies have found an association between stress at work and physical inactivity [4,12,18-20]; others found this association to be weak [21] or absent [22,23]. Although the association between job strain and smoking cessation has been previously demonstrated [16,24], the results of studies on the work environment and smoking have been even more inconclusive—especially with regard to the amount smoked [11,16,23,25-27]. Less study has been devoted to the putative effect of social support on those behaviors [16,22]. What has also been shown, albeit less frequently, is that the effects of job strain and health-related behavior differ between men and women [18,20,22,28-30].

Most studies on the association between job strain and health behavior have been conducted in Europe or North America. To our knowledge, few investigations have examined this association in Latin America, where the work context is markedly different [17,23]. The present study examined the association between psychosocial job strain and two behaviors—smoking and leisure-time physical inactivity—that increase the risk of chronic diseases in currently employed participants in the baseline assessments of the Brazilian Longitudinal Study of Adult Health (ELSA-Brasil).

## Methods

### Study design

ELSA-Brasil is a multicenter study involving universities and research institutes in six Brazil’s states (São Paulo, Minas Gerais, Rio Grande do Sul, Bahia, Espírito Santo, and Rio de Janeiro). The main goal of the study is to determine the incidence of cardiovascular diseases and their biological and social determinants in a Brazilian context. Details of the study design, including eligibility criteria and recruitment methods, have already been published [31]. Basically, all active or retired employees of the six institutions of either sex and aged 35–74 years were eligible for the study. Exclusion criteria were as follows: (1) current or recent (<4 months prior to the first interview) pregnancy; (2) intention to quit working at the institution in the near future; (3) severe cognitive or communication impairment; and (4) if retired, residence outside a study center’s corresponding metropolitan area [31]. Efforts were made to recruit similar proportions of men and women as well as predefined proportions of age-groups and occupational categories [31].

From a total of 16,435 individuals who expressed interest in participation, 15,821 were pre-enrolled, provided their written consent, responded to an initial interview, and were scheduled for the baseline examination conducted from 2008 to 2010. Only 716 (4.5%) of those pre-enrolled failed to complete the baseline examination. The baseline study comprised 15,105 active and retired civil servants, whose state of health is being monitored on an ongoing basis. Thereupon, an evaluation lasting about 7 hours was conducted; it included a face-to-face interview, clinical examinations, and anthropometric measurements, and it followed a rigorous process to guarantee and control quality [32]. Of 12,096 current workers in the ELSA-Brasil baseline assessment, the following were excluded: those lacking information for the job-stress scale; those who did not answer questions relating to physical activities or smoking. Accordingly, this study analyzed the data of 11,779 individuals (97.3%; 5,625 men, 6,154 women) with regard to job strain and leisure-time physical activity and 11,963 individuals (98.9%; 5,712 men, 6,251 women) with regard to job strain and smoking.

### Exposure—psychosocial job strain

The exposure variable was obtained using the Brazilian version of the Swedish Demand-Control-Support Questionnaire (DCSQ) [33], which is based on the Job Content Questionnaire (JCQ) [34]. Like the JCQ, the DCSQ comprises three dimensions—psychological demands (five items), control (six items), and social support (6 items)—with psychometric properties appropriate for Brazil [35]. The DCSQ questionnaire is considered reliable and correlates highly with the JCQ [34]. Compared with the JCQ, the DCSQ has the following features: (1) a small number of items; (2) a response grading based on frequency, whereas the JCQ considers intensity; and (3) support items are oriented toward the atmosphere at the worksite—in the JCQ, the items are based on more objective and instrumental aspects of relationships with coworkers and supervisors [34,36].

We evaluated the continuous scores for every dimension separately. In addition, in accordance with the model proposed by Karasek [9] for studying job strain, values in each dimension were categorized by the median as low (≤median) or high (>median), which yielded combinations of low or high demand and low or high control. The median values for demand and job control were 14 and 18, respectively. From that, we were able to construct the quadrants: low-strain (low demand/high control; reference category), passive work (low demand/low control), active work (high demand/high control) and high-strain (high demand/low control).

### Outcomes—physical inactivity and smoking

We measured the time spent on leisure-time physical activity by means of the International Physical Activity Questionnaire (IPAQ-short) [37]. The instrument inquires how many times a week respondents engage in walking and moderate or vigorous physical activities for at least 10 minutes in their free time as well as how long on any given day. For the purposes of analysis, the physical activity measure was obtained by multiplying the number of days on which physical activity was undertaken by the duration in minutes. Participants were then classified into three frequency levels—none, <150 minutes/week, and ≥150 minutes/week (reference category)—in line with international recommendations on physical activity to promote and maintain health in adults [38].

Smoking was determined by self-report if the participants declared having smoked at least 100 cigarettes in their lifetime. It was categorized into “non-smokers” (reference category including those who had never smoked and former smokers) and “current smokers” (those who still smoked at the time of research). For current smokers, we measured the level of exposure to smoking by the number of cigarettes smoked per day.

### Covariates

Socioeconomic and demographic variables applied as covariates in the models were as follows: age (continuous); schooling (never attended school or incomplete elementary education, completed elementary school, high school, university degree, and postgraduate); hours worked per week (continuous); and standardized per capita family income (continuous), calculated from the midpoint of the category of reported net income divided by the number of dependents on that income. Owing to major variations, the latter variable was standardized by reducing each value by the mean and dividing by the standard deviation. All these variables were evaluated as possible confounders because they were associated with job strain and also influenced both leisure-time physical activity and smoking status.

### Statistical analyses

We estimated the association between exposure variables and categorical outcomes of interest using multiple logistic regression analysis (for smoking) and multinomial logistic regression (for physical activity). The association between exposure variables and number of cigarettes smoked was evaluated using a negative binomial regression model because of the dispersion of the number of cigarettes variable [39]. Odds ratios and 95% confidence intervals were estimated from multiple models. Previous tests using analysis of variance showed collinearity between income and education (*p* <0.0001), and only the latter was used in the regression models. Crude and adjusted associations were demonstrated (model 1, crude; model 2, adjusted for age and marital status; model 3, adding the variable of educational level; and model 4, adding the variable of hours worked weekly). We used the Akaike Information Criterion for comparison between models, and lower values were considered best fitted [40].

We performed all the analyses separately by sex. Interaction tests were conducted to examine the influence of the number of hours worked weekly on the association between psychosocial job strain and evaluated behaviors. All the analyses used a 0.05 level of significance. The analyses were performed with the VGAM library of R, version 3.02 [41].

### Ethical considerations

All participants signed a letter indicating their free and informed consent. The study was approved by the National Research Ethics Commission (*Comissão Nacional de Ética em Pesquisa*, CONEP; No. 976/2006) and also by the Research Ethics Committee of each institution: Hospital Universitário da Universidade de São Paulo (Universidade de São Paulo), Fundação Oswaldo Cruz (Fundação Oswaldo Cruz), Instituto de Saúde Coletiva da Universidade Federal da Bahia (Universidade Federal da Bahia), Universidade Federal de Minas Gerais (Universidade Federal de Minas Gerais), Centro de Ciências da Saúde da Universidade Federal do Espírito Santo (Universidade Federal do Espírito Santo) and Hospital de Clínicas de Porto Alegre (Universidade Federal do Rio Grande do Sul).

## Results

At study baseline, the mean age of the population was 49.2 years, and it comprised similar proportions of men (47.8%) and women (52.2%). Notably, 52.8% of participants held a university degree, 67.5% were married, 44.5% engaged in no leisure-time physical activity, and 13.6% were smokers (mean of 12.4 cigarettes per day).

Men and women with less income and schooling and those classified as in high-strain and passive-work jobs reported higher frequencies of physical inactivity in their leisure time. Smoking was reported more frequently by workers with less income and schooling, those separated or widowed, and also those classified as in active and high-strain jobs (Tables 1 and 2).

**Table 1:** **Prevalence of leisure-time physical activity among men and women by variables examined, ELSA-Brasil, baseline (2008–10)**

**Variables**	**Men (n = 5625)**			**Women (n = 6154)**		
	**None**	**<150 min/week**	**≥150 min/week**	**None**	**<150 min/week**	**≥150 min/week**
	**(n = 2158)**	**(n = 1305)**	**(n = 2162)**	**(n = 3083)**	**(n = 1271)**	**(n = 1800)**
**Mean age** **(SD)**	49.6 (7.3)	49.7 (7.6)	49.5 (7.4)	48.7 (7.0)	49.2 (7.2)	49.0 (7.1)
**Income, terciles** **Mean (SD)**	1287.28(1027.6)	1553.55(1197.5)	1795.11(1385.0)***	1372.0(1094.2)	1854.9(1442.8)	1996.6(1443.3)***
**Schooling**						
Never attended school or incomplete elementary school	57.4	21.3	21.3***	71.7	13.9	14.5***
Complete elementary school	46.3	23.3	30.4	66.3	16.8	16.8
High school	44.3	21.9	33.8	60.8	17.3	21.9
University degree	38.6	23.1	38.3	47.9	21.3	30.8
Postgraduate	27.4	24.8	47.8	36.7	24.7	38.6
**Marital status**						
Married	38.8	23.8	37.3**	50,5	21,2	28,3
Separated or widowed	35.9	21.1	43.0	50,3	19,9	29,8
Single	37.5	19.2	43.3	48,2	20,1	31,8
**Weekly hours worked** **Mean (SD)**	44.1(11.8)	45.4 (11.1)	44.6 (10.9)***	44.1 (10.0)	45.4 (10.1)	44.6 (10.4)***
**Job strain**						
Low D/High C (low strain)	33.5	22.4	44.2*	40.9	23.7	35.4**
High D/High C (active work)	30.7	25.9	43.4	41.1	22.8	36.1
Low D/Low C (passive work)	44.5	21.7	33.9	57.1	18.5	24.4
High D/Low C (high strain)	43.2	25.4	31.4	56.5	19.0	24.5
**Demand** **Mean (SD)**	12.9 (2.9)	13.3 (2.7)	13.1 (2.7)**	13.5 (2.9)	13.7 (2.7)	13.7 (2.8)*
**Control** **Mean (SD)**	17.6 (2.9)	18.2 (2.9)	18.6 (2.9)**	17.1 (2.9)	17.9 (2.9)	18.3 (2.9)*
**Social support at work** **Mean (SD)**	20.1 (3.3)	19.8 (3.2)	19.9 (3.2)	19.5 (3.3)	19.5 (3.2)	19.6 (3.2)

*p < 0.05; **p < 0.010; ***p < 0.001.

**Table 2:** **Prevalence of smoking among men and women by variables examined, ELSA-Brasil, baseline (2008–10)**

**Variables**	**Men (n = 5712)**		**Women (n = 6251)**	
	**Non-smokers (n = 4865)**	**Smokers (n = 847)**	**Non-smokers (n = 5468)**	**Smokers (n = 783)**
**Mean age** **(SD)**	49.5 (7.6)	49.8 (6.6)	48.8 (7.2)	49.6 (6.0)***
**Income, terciles** **Mean (SD)**	1579.9(1245.4)	1310.7(1122.6)***	1669.4(1304.7)	1515.2(1322.9)***
**Educational level**				
Never attended school or incomplete elementary school	73.6	26.4***	80.3	19.7***
Complete elementary school	76.9	23.1	79.1	20.9
High school	81.8	18.2	83.8	16.2
University degree	91.3	8.7	91.3	8.7
Postgraduate	88.3	11.7	90.3	9.7
**Marital status**				
Married	86.1	13.9***	89.6	10.4***
Separated or widowed	79.1	20.9	82.7	17.3
Single	86.8	13.2	89.7	10.3
**Weekly hours worked** **Mean (SD)**	44.8 (11.4)	43.3 (10.5)***	42.1 (10.2)	41.8 (9.6)
**Job strain**				
Low D/High C (low strain)	87.3	12.7***	88.5	11.5*
High D/High C (active work)	82.5	17.5	87.4	12.6
Low D/Low C (passive work)	89.2	10.8	88.7	11.3
High D/Low C (high strain)	82.3	17.7	85.3	14.7
**Demand** **Mean (SD)**	13.1 (2.8)	12.9 (2.8)	13.5 (2.9)	13.6 (3.2)
**Control** **Mean (SD)**	18.2 (2.9)	17.6 (3.0)***	17.7 (2.9)	17.3 (3.1)**
**Social support at work** **Mean (SD)**	20.0 (3.2)	19.8 (3.4)	19.5 (3.2)	19.2 (3.4)

*p < 0.05; **p < 0.010; ***p < 0.001.

Crude analyses of the association between psychosocial job strain and physical activity showed greater likelihood of physical inactivity, or activity for shorter duration than recommended, among men classified in the high-strain and passive-work quadrants. Among women, higher likelihood of physical inactivity was observed among those classified as having passive-work and high-strain jobs.

After adjustment, men in high-strain and passive-work jobs had, respectively, 34% (95% confidence interval [CI] = 1.09–1.64) and 22% (95% CI = 1.03–1.43) greater likelihood of not engaging in physical activity than those doing low-strain work. In addition, men in high-strain jobs had 41% (95% CI = 1.15–1.82) greater likelihood of exercising for less time than recommended. Higher probability of physical inactivity was observed among women in high-strain (odds ratio [OR] = 1.47; 95% CI = 1.22–1.77) and passive-work jobs (OR = 1.42; 95% CI = 1.20–1.67; Table 3). The interaction terms indicated no influence of work hours on the association between job stain and leisure-time physical activity (*p* = 0.124 and *p* = 0.644, respectively, for men and women). With regard to separate dimensions, job control was protective against physical inactivity in both sexes (men, OR = 0.96, 95% CI = 0.93–0.98; women, OR = 0.93, 95% CI = 0.91–0.96). Among women, social support was also observed to have a protective effect against physical inactivity (OR = 0.98; 95% CI = 0.96–0.99; Table 3), but the association was no longer significant after adjustment for the other dimensions (psychological demands and control at work). No association was observed between psychological demands and leisure-time physical activity.

**Table 3:** **Crude and adjusted odds ratios of the association between psychosocial job strain (quadrants and dimensions separate) and physical activity among men and women, ELSA-Brasil, baseline (2008–10)**

**Models**	**Men (n = 5625)**		**Women (n = 6154)**	
	**None**	**<150 min/week**	**None**	**<150 min/week**
	**OR (CI95%)**	**OR (CI95%)**	**OR (CI95%)**	**OR (CI95%)**
***Model 1: crude***				
Low D/High C (low strain)	1.00	1.00	1.00	1.00
High D/High C (active work)	0.94 (0.78-1.12)	1.18 (0.97-1.43)	0.98 (0.82-1.17)	0.94 (0.77-1.15)
Low D/Low C (passive work)	1.73 (1.50-2.01)***	1.27 (1.07-1.50)**	2.02 (1.73-2.36)***	1.13 (0.94-1.36)
High D/Low C (high strain)	1.82 (1.50-2.21)***	1.60 (1.28-1.99)***	1.99 (1.67-2.38)***	1.16 (0.93-1.44)
**Model 2: age, marital status**				
Low D/High C (low strain)	1.00	1.00	1.00	1.00
High D/High C (active work)	0.94 (0.78-1.12)	1.18 (0.97-1.44)	0.98 (0.83-1.17)	0.94 (0.77-1.16)
Low D/Low C (passive work)	1.74 (1.51-2.02)***	1.28 (1.07-1.51)**	2.04 (1.75-2.39)***	1.14 (0.94-1.37)
High D/Low C (high strain)	1.83 (1.51-2.23)***	1.61 (1.29-2.01)***	2.00 (1.67-2.38)***	1.18 (0.95-1.46)
**Model 3: age, marital status, educational level**				
Low D/High C (low strain)	1.00	1.00	1.00	1.00
High D/High C (active work)	1.00 (0.84-1.21)	1.21 (0.99-1.47)	1.05 (0.88-1.26)	0.95 (0.78-1.17)
Low D/Low C (passive work)	1.20 (1.02-1.41)*	1.13 (0.94-1.37)	1.42 (1.20-1.67)***	1.06 (0.87-1.29)
High D/Low C (high strain)	1.36 (1.11-1.67)**	1.48 (1.18-1.86)***	1.47 (1.22-1.77)***	1.11 (0.89-1.39)
**Model 4: age, marital status, educational level and weekly hours worked**				
Low D/High C (low strain)	1.00	1.00	1.00	1.00
High D/High C (active work)	0.96 (0.80-1.16)	1.15 (0.94-1.40)	1.05 (0.87-1.25)	0.93 (0.76-1.15)
Low D/Low C (passive work)	1.22 (1.03-1.43)*	1.15 (0.95-1.38)	1.42 (1.20-1.67)***	1.06 (0.87-1.30)
High D/Low C (high strain)	1.34 (1.09-1.64)**	1.44 (1.15-1.82)**	1.47 (1.22-1.77)***	1.10 (0.88-1.38)
**Model dimensions**				
**Psychological demands**				
Model 1: crude	0.98 (0.96-1.00)	1.03 (1.00-1.05)*	0.97(0.95-0.99)*	1.00(0.98-1.03)
Model 2: Model 1 + age and marital status	0.98 (0.96-1.00)	1.03 (1.00-1.05)*	0.97(0.95-0.99)*	1.00(0.98-1.03)
Model 3: Model 2 + educational level	1.01 (0.99-1.03)	1.04 (1.01-1.06)**	1.00(0.98-1.03)	1.01(0.98-1.04)
Model 4: Model 3 + weekly hours	1.00 (0.98-1.02)	1.03 (1.00-1.06)*	1.00(0.98-1.03)	1.01(0.98-1.03)
Model 5: Model 4 + control and social support at work	1.00 (0.98-1.02)	1.03 (1.00-1.05)*	1.00(0.98-1.03)	1.01(0.98-1.04)
**Control at work**				
Model 1: crude	0.90 (0.88-0.91)***	0.96 (0.94-0.98)***	0.87(0.85-0.89)***	0.96(0.93-0.98)**
Model 2: Model 1 + age, marital status	0.90 (0.88-0.91)***	0.96 (0.94-0.98)***	0.87(0.85-0.89)***	0.96(0.93-0.98)**
Model 3: Model 2 + educational level	0.96 (0.94-0.98)***	0.98 (0.95-1.01)	0.93(0.91-0.95)***	0.97(0.94-1.00)*
Model 4: Model 3 + weekly hours	0.96 (0.93-0.98)***	0.98 (0.95-1.00)	0.93(0.91-0.95)***	0.97(0.94-0.99)*
Model 5: Model 4 + psychological demands and social support at work	0.96 (0.93-0.98)***	0.98 (0.95-1.01)	0.93(0.91-0.96)***	0.97(0.94-1.00)*
**Social support at work**				
Model 1: crude	1.01 (0.99-1.03)	0.99 (0.97-1.01)	1.00(0.98-1.01)	0.99(0.97-1.02)
Model 2: Model 1 + age, marital status	1.01 (0.99-1.03)	0.99 (0.97-1.01)	1.00(0.98-1.01)	0.99(0.97-1.01)
Model 3: Model 2 + educational level	0.98 (0.96-1.00)	0.98 (0.96-1.00)	0.98(0.96-0.99)*	0.99(0.96-1.01)
Model 4: Model 3 + weekly hours	0.98 (0.96-1.00)	0.98 (0.96-1.00)	0.98(0.96-0.99)*	0.99(0.97-1.01)
Model 5: Model 4 + psychological demands and control at work	0.99 (0.97-1.01)	0.99 (0.97-1.01)	0.99 (0.97-1.01)	0.99 (0,97-1.02)

reference category: physical activity ≥ 150 min/week.

*p < 0.05; **p < 0.010; ***p < 0.001.

OR, Odds ratio; CI95%, 95% confidence interval.

Regarding the association between job strain quadrants and smoking, the crude associations observed among men and women ceased to be significant after adjustment for educational level. The interaction terms indicated no effect of work hours on the association between job stain and smoking status (*p* = 0.340 and *p* = 0.394, respectively, for men and women). However, after all adjustments, including demands, control and support, the likelihood of women and men being smokers decreased, respectively, by 4% (OR = 0.96; 95% CI = 0.94–0.98) and 5% (OR = 0.95; 95% CI = 0.93–0.98) with each one-point increase in the score for social support (Table 4). No association was found between psychosocial job strain (quadrants and dimensions) and number of cigarettes smoked per day (data not shown).

**Table 4:** **Crude and adjusted odds ratios of the association between psychosocial job strain (quadrants and dimensions separate) and smoking among men and women, ELSA-Brasil, baseline (2008–10)**

**Model quadrants**	**Men (n = 5712)**	**Women (n = 6251)**
	**OR (95%CI)**	**OR (95%CI)**
**Model 1: crude**		
Low D/High C (low strain)	1.00	1.00
High D/High C (active work)	0.83 (0.66-1.06)	0.99 (0.78-1.26)
Low D/Low C (passive work)	1.47 (1.23-1.76)***	1.11 (0.91-1.36)
High D/Low C (high strain)	1.49 (1.18-1.87)***	1.33 (1.06-1.66)
**Model 2: age, marital status**		
Low D/High C (low strain)	1.00	1.00
High D/High C (active work)	0.84 (0.66-1.07)	0.99 (0.78-1.26)
Low D/Low C (passive work)	1.49 (1.24-1.78)***	1.11 (0.91-1.36)
High D/Low C (high strain)	1.50 (1.19-1.89)***	1.36 (1.09-1.70)
**Model 3: age, marital status and educational level**		
Low D/High C (low strain)	1.00	1.00
High D/High C (active work)	0.90 (0.71-1.16)	1.04 (0.82-1.33)
Low D/Low C (passive work)	0.98 (0.81-1.20)	0.81 (0.65-1.01)
High D/Low C (high strain)	1.08 (0.85-1.37)	1.03 (0.81-1.30)
**Model 4: age, marital status, educational level and weekly hours worked**		
Low D/High C (low strain)	1.00	1.00
High D/High C (active work)	0.91 (0.71-1.16)	1.02 (0.80-1.30)
Low D/Low C (passive work)	0.98 (0.81-1.20)	0.81 (0.65-1.01)
High D/Low C (high strain)	1.08 (0.85-1.37)	1.02 (0.81-1.29)
**Model dimensions**		
**Psychological demands**		
Model 1: crude	0.97 (0.95-1.00)*	1.01 (0.98-1.04)
Model 2: Model 1 + age and marital status	0.98 (0.95-1.00)	1.01 (0.99-1.04)
Model 3: Model 2 + educational level	1.00 (0.98-1.03)	1.03 (1.01-1.06)*
Model 4: Model 3 + weekly hours	1.00 (0.98-1.03)	1.03 (1.00-1.06)*
Model 5: Model 4 + control and social support at work	0.99 (0.96-1.02)	1.02 (0.99-1.06)
**Control at work**		
Model 1: crude	0.93 (0.91-0.95)***	0.96 (0.94-0.99)**
Model 2: Model 1 + age, marital status	0.93 (0.91-0.95)***	0.96 (0.94-0.99)**
Model 3: Model 2 + educational level	1.00 (0.97-1.03)	1.02 (0.99-1.05)
Model 4: Model 3 + weekly hours	1.00 (0.98-1.03)	1.02 (0.99-1.05)
Model 5: Model 4 + psychological demands and social support at work	1.01 (0.98-1.04)	1.03 (1.00-1.06)
**Social support at work**		
Model 1: crude	0.98 (0.96-1.00)	0.97 (0.95-0.99)*
Model 2: Model 1 + age, marital status	0.98 (0.96-1.00)	0.97 (0.95-0.99)**
Model 3: Model 2 + educational level	0.96 (0.94-0.98)***	0.96 (0.94-0.98)***
Model 4: Model 3 + weekly hours	0.96 (0.94-0.98)***	0.96 (0.94-0.98)***
Model 5: Model 4 + psychological demands and control at work	0.95 (0.93-0.98)***	0.96 (0.94-0.98)***

reference category: non-smokers.

*p < 0.05; **p < 0.010; ***p < 0.001.

OR, Odds ratio; 95%CI, 95% confidence interval.

## Discussion

The results indicate that the high-strain and passive-work groups were more likely to be physically inactive in their leisure time or engage in physical activities for less than the recommended amount; however, patterns differed between men and women. Among women, both situations were associated with physical inactivity only; among men, high strain was associated with both physical inactivity and with physical activity for less than the recommended time to promote health. High-strain work is recognized as a risk factor for physical inactivity, although the association is generally weak [4,20,21,42]; the association may differ by socioeconomic status [18]. Among women, high levels of strain at work can have even more substantial effects on physical inactivity if combined with the demands of family responsibilities [19,43].

Our results appear to corroborate the theory of Karasek [9] with regard to passive behaviors in various spheres of life (at work and outside) among the passive-work group. Others authors have found similar results to ours for men [44] and for both sexes [19]. Artazcoz and collaborators [43] found that working conditions had greater impact on men’s health, while domestic demands were more important among women. Although not explored in the present study, the domestic work overload identified among women in Brazil [45,46] may have interacted with the characteristics of professional work and affected the association between passive work and physical inactivity among women. In addition, lack of time is reported to be one the reason for not engaging in healthy behaviors, such as physical exercise and healthy eating [47]. It could be argued that those with active jobs have more control over their work time and time in general, which makes it easier to undertake physical exercise, than those with job strain and perhaps also low control over work time.

In both sexes, higher levels of job control were independently associated with the lowest likelihood of leisure-time physical inactivity, even when adjusted for psychological demands and social support at work; however, for psychological job demands, we did not observe this association. Other studies have obtained similar results [18,19,48]. Smith et al. [48] suggest that both the diffusion of lack of job control into daily life and lack of time to plan opportunities to participate in leisure-time physical activity make participation in physical activities more challenging for workers with little control over their jobs. In addition, it can be speculated that the advantageous situation of having control over working hours—allied to greater social prestige and the context of very stable employment (as is the case with civil servants at Brazilian universities)—is reflected in how individuals deal with health-related factors as well as with the opportunity to engage in leisure-time physical activities.

After adjustments, no association was observed between the quadrants or their respective dimensions and smoking. Psychosocial factors have probably declined in influence under the strong influence of public policies designed to control smoking in Brazil; those policies have led to widespread cessation of smoking in recent years [49]. Although levels of schooling were higher in this study population than in Brazil overall, the social gradient of smoking is still substantial, which points to a stronger association with schooling and income than with psychosocial job strain. Other authors [25] have identified a weak association between smoking and the components of psychosocial job strain. Smoking generally starts before entry into the labor market, and whether or not it is maintained depends on a range of social and cultural factors [25,50]. Some authors [16,25] suggest that job strain influences the intensity and continuance of smoking, rather than making a difference between being or not being a smoker. Our results do not confirm this hypothesis with regard to the number of cigarettes smoked. Longitudinal studies will be able to assess the influence of job strain on whether or not the habit of smoking is maintained.

The present study found that among both men and women, social support at work reduced the likelihood of smoking, whereas among women it reduced the likelihood of physical inactivity. Since this study population was aged 35–74 years, in very stable employment, and, accordingly, had worked for a long period at their institutions (mean duration of work at the institution was 20.2 ± 9.5 and 19.1 ± 8.8 years for men and women, respectively), those circumstances may have underscored the importance of social support networks at their workplace. It is notable that most studies that have evaluated how psychosocial job strain is associated with smoking and physical activity have not examined the workplace social support component [13,18,20,21,27-29,51]; that limits the comparisons with the results of the present investigation. Some studies found a positive association among social support at work and smoking cessation, relapse, and the amount smoked [16,24]. However, it is suggested in the literature that social support in life (not necessarily at work) is related to smoking cessation and maintenance of cessation [52,53] and to engagement in and maintenance of physical activity [54]. Therefore, subsequent studies should take account of this dimension.

### Strengths and limitations

This investigation presents the results based on detailed data carefully collected from almost 12,000 current workers of the ELSA-Brasil study, which was conducted in six Brazilian cities. To the best of our knowledge, this is the first large-scale study to examine job strain in relation to insufficient leisure-time physical activity and smoking in Brazil. However, some limitations deserve attention. This is a cross-sectional study, and temporality cannot be established. Nevertheless, reverse causality seems unlikely in physical inactivity—as demonstrated in a recent systematic review [55] and longitudinal study [18]; consistent with the expected direction of causality, those reports found that job strain predicts physical inactivity more strongly than physical inactivity can predict strain. With smoking, the temporal evidence on the nature of the association is less clear, and the associations may possibly be bidirectional and explainable by common causes [18,27]. Although ELSA-Brasil addressed a rather specific population, its results reflect the situation for a portion of Brazil’s population in employment and residing in cities. In addition, the influence of other factors that could be considered confounding (for example, alcohol consumption and physical effort at work) was not addressed, and they could have attenuated the observed associations.

## Conclusions

Our findings point to the importance of psychosocial job strain in physical activity and of social support in smoking. These results provide additional evidence for the association between the psychosocial work environment and health-related behaviors, and our findings may be of benefit in workplace health planning and promotion. For that reason, the work environment should be considered in health-care policies designed to promote healthier habits in the population of an economically active age.
